# Sexual and gender minorities rights in Latin America and the Caribbean: a multi-country evaluation

**DOI:** 10.1186/s12914-019-0217-3

**Published:** 2019-11-06

**Authors:** Monica Malta, Reynaldo Cardoso, Luiz Montenegro, Jaqueline Gomes de Jesus, Michele Seixas, Bruna Benevides, Maria das Dores Silva, Sara LeGrand, Kathryn Whetten

**Affiliations:** 1 Faculty of Medicine, Department of Psychiatry, University of Toronto, Toronto, 33 Russell Street / Room RS 2035, Toronto, Ontario M5S 2S1 Canada; 2 Institute for Mental Health Policy Research, Centre for Addiction and Mental Health, Toronto, ON Canada; 3 Department of Social Science, National School of Public Health, Oswaldo Cruz Foundation (ENSP/FIOCRUZ), Rio de Janeiro, Brazil; 4 Department of International Law, Organization of American States (OAS), Washington, DC USA; 5 Department of Epidemiology and Quantitative Methods in Health, Sergio Arouca National School of Public Health (DEMQS-ENSP), FIOCRUZ, Rio de Janeiro, Brazil; 6 Rio de Janeiro Federal Institute of Education, Science and Technology, Belford Roxo, RJ Brazil; 7 UN Women Brazil, Civil Society Advisory Group, Brasilia, DF Brazil; 8 Felipa de Sousa Women’s Group, Rio de Janeiro, RJ Brazil; 9 Brazilian National Association of Transgender and Travesties (ANTRA), Salvador, BA Brazil; 10 Department of Social Science, Sergio Arouca National School of Public Health (DCS-ENSP), FIOCRUZ, Rio de Janeiro, Brazil; 11 Center for Health Policy and Inequalities Research, Duke Global Health Institute, Duke University, Durham, NC USA

## Abstract

**Background:**

Although the extent of legal inequities experienced by sexual and gender minorities (SGM) has declined during recent decades, this population still enjoys fewer legal protections and benefits than the non-gender-variant, heterosexual population. Herein we analyze the current scenario of SGM rights in Latin America and the Caribbean (LAC).

**Methods:**

Policy documents and governmental strategies addressing SGM rights were analyzed within a timeline framework by three major LAC sub-regions: the Caribbean, Mesoamerica and South America.

**Results:**

Our search identified 88 eligible documents addressing the following categories: (1) legal protections towards same-sex couples (decriminalization of same-sex acts among consenting adults, legal recognition of same-sex unions, same-sex marriage, adoption by same-sex couples), and (2) anti-discrimination laws (SGM allowed to serve openly in the military and anti-discrimination laws related to sexual orientation, gender identity and/or expression). The majority of Caribbean countries prohibit same-sex acts between consenting adults, while in Mesoamerica same-sex couples do not have equal marriage rights and are not allowed to adopt as a couple. In the Caribbean and Mesoamerica transgender people lack proper legal protection. Legislation to protect SGM rights in South America is the most inclusive and progressive in LAC. Several countries recognize same-sex marriage and the right of transgender people to legally change their name and gender. The majority of South American countries have some kind of anti-discrimination law, but no effective mechanisms to enforce these laws. In spite of those progresses, the LAC region registers the highest rate of violence and hate crimes against SGM in the world.

**Conclusion:**

In the Caribbean and Mesoamerica the overall discriminatory legislation exacerbates violence against SGM within a social and cultural context of strong sexist, gender stereotypes and widespread violence. This scenario is driving hundreds of SGM to leave their home countries. In spite of progressive legislations, several South American countries are currently controlled either by highly conservative leaders (e.g. Brazil and Chile) or by repressive dictators (Venezuela). The near future of the LAC region is unknown, but if such trends continue, severe human rights problems, including setbacks in SGM legal protections, are likely.

## Background

According to the Commission on Social Determinants of Health, from the World Health Organization, “social justice is a matter of life and death. It affects the way people live, their consequent chance of illness, and their risk of premature death” [1–3].

The lack of social justice and frequent social stressors (e.g. victimization and discrimination) affect health inequities frequently identified among sexual and gender minorities – SGM [4]. Sexual minority women seem to have greater risk for breast cancer and cardiovascular disease relative to heterosexual women [5, 6], while sexual minority men have increased risk for sexually transmitted infections compared to heterosexual men [7]. SGM have higher rates of smoking and heavy drinking than the general population [6, 8]. Depression and anxiety disorders are more prevalent among transgender people [9–11] and sexual minorities [6] when compared to the general population.

In spite of those increased health risks, SGM are less likely to access the care they need [11–14], when compared to non-SGM groups. Those inequities in healthcare provision are mostly influenced by stigma and discrimination [15, 16], lack of sufficient health insurance [17], inadequate knowledge or prejudice by healthcare providers [18].

One of the most prominent framework to study SGM health inequities is the minority stress model [19]. This theoretical model addresses the relationship between minority and dominant values in society and resultant conflicts with the social environment that are experienced by minority group members [19–21]. The impact of increased and frequent exposure of SGM to stress, when compared to heterosexual cisgender individuals, highly influences SGM health inequities [22]. Although the extent of legal inequities experienced by SGM has declined in many countries during recent decades, this population still enjoys fewer overall legal rights, protections, and benefits than the non-gender-variant, heterosexual population. Inequities in the law can affect fundamental aspects of SGM lives and might influence their health and ability to access quality health care [23, 24].

For instance, in countries that do not authorize same-sex civil unions or marriage, same-sex couples are denied a variety of benefits. In many cases, employer-sponsored health insurance is not extended to same-sex partners, affecting their access to affordable health care. In addition, SGM are often unable to request medical leave from work to care for an unmarried same-sex partner to the same extent as married couples [25].

The lack of recognition of same-sex civil unions or marriage has other health implications as well. Some studies have identified positive health outcomes associated with same-sex marriage [26, 27]. Riggle et al. [28] conducted a study with 2677 lesbian, gay, and bisexual adults, and identified that same-sex couples in legally recognized relationships experienced fewer depressive symptoms, lower levels of stress and reported less internalized homophobia, when compared with long-term same-sex partners without legal recognition.

Discriminatory polices deeply affect transgender people lives, their health status and healthcare access in numerous ways [29]. For example, legal recognition of one’s gender identity is not only essential for a person’s dignity, but also necessary to ensure access to the most basic services, including legal identification, formal education, employment, social security, health services, public safety and legal protection [16, 30, 31]. A lack of documents that match an individual’s gender identity can result in denial of health and social support services, travel restrictions, bullying, humiliation, and gender-based violence [30, 32, 33].

Transgender individuals encounter unique barriers to access healthcare, when compared to sexual minority men and women, a disparity closely related to legal inequities [33]. Inadequate legislations could jeopardize transgender individuals’ access to hormone treatment and gender-affirming surgical procedures, frequently affecting their quality of life and mental health [34].

SGM rights are more likely to advance in democracies where social movements are strong, organized, sufficiently networked, and where religion is less influential in daily life and policy development [35]. However, even strong democracies with well-organized social movements can experience setbacks under highly conservative governmental administrations. For instance, the Trump administration recently removed sexual orientation and gender identity questions from national surveys [36], has banned transgender recruits from joining the military and is revising anti-discrimination regulations – a decision that could decrease SGM access to healthcare, government-funded shelters, economic and social benefits [12, 13, 37].

In Latin America and Caribbean (LAC) countries, SGM rights have developed in close synchrony with growing social movements [38]. Fights for democratization, after years of dictatorship, presented an important juncture for social movements in several LAC countries [39]. Transition to democratization dramatically changed the political opportunities in LAC countries, which allowed for a different type of interaction between the state and social movements [40].

However, even after the return to democracy, SGM did not have any civil, political or social rights. The re-emergence of activism was related, in part, to continued rights-based oppression of SGM under democratically elected governments. For example, in terms of civil rights, three months after Raul Alfonsin was sworn in as President of Argentina, “approximately 50 activists were detained in a gay club” [39]. As for political rights, “up until 1990, a law (albeit) unenforced was on the books in Buenos Aires” that essentially banned SGM from voting [40].

In Brazil, the role of social movements during the democratic transition was crucial for ousting the military dictatorship that ruled the country from 1964 to 1985 [41]. During the 1980’s the SGM community mainly organized itself to fight the AIDS epidemic, while actively participating in broader discussions and manifestations towards democracy, human rights and health sector reform [42]. However, only in 2013 the country adopted a National Comprehensive Health Plan addressing SGM needs and specificities [43].

Evaluations of major gaps in SGM-related legislation and discriminatory laws can inform policy reforms and guide changes on licensing boards, ethics committees, and other regulatory bodies. The collaborative effort of activists, researchers, health professionals and policy analysts have been pivotal to identify the harmful outcomes of “conversion therapies”, informing legislations that ban these “therapies” [44]. However, assessing changes in the legal environment and how it might affect SGM’s quality of life is challenging. The causal pathway is frequently long and complex, large-scale changes in legislation do not occur quickly, and there is no gold standard methodology.

Outside of high-income countries, no region has experienced more progress in expanding SGM rights than Latin America [38–40]. While many SGM rights in the United States still involve some degree of legal dispute, in several countries in Latin America laws about same-sex marriage and adoption, changing gender on national ID cards, and anti-discrimination laws started in the past decade - many before the US Supreme Court legalized same-sex marriage [45–47].

Nevertheless, worldwide, the highest rate of violence against SGM is found in the LAC region [38]. This article explores this paradigm by analyzing the current scenario of SGM rights in LAC, key successes, major gaps and future challenges.

## Methods

We documented and compared policies addressing SGM rights in LAC countries, following James Mahoney’s sociological framework of ‘policy path dependency’ [48]. This framework allows for the identification of policies based on chronological order, enabling the visualization of changes across countries over time. We hypothesized that previous decisions and policy frameworks could influence changes in future SGM-related legislations. We also assumed the path dependency to follow a ‘reactive sequence’, instead of a ‘self-reinforcing’ sequence. According to Mahoney [48], ‘self-reinforcing’ sequences are characterized by successive events that reinforce early events, while ‘reactive sequences’ are marked by backlash processes that transform and sometimes reverse previous events – therefore stimulating policy changes over time through a chain or path of events linked by reactions and counter-reactions. According to Paul Pierson, “initial disturbances are crucial not because they generate positive feedback, but because they trigger a powerful response ( …) where action and reaction shift the system in a new direction, but not one that reinforces the first move” [49].

This methodology is suited to explore policy pathways based on precedent legislations and is particularly adequate to understand how (and if) laws are changed in response to human rights violations. We considered three structural documents in our analysis: the landmark Universal Declaration of Human Rights – UDHR [50], adopted by the UN General Assembly in 1948, as well as the International Covenant on Civil and Political Rights (ICCPR) and the International Covenant on Economic, Social and Cultural Rights (ICESCR), both adopted by the UN General Assembly in 1966 [51, 52]. These three documents — the UDHR, ICCPR, and ICESCR — are referred collectively as the “International Bill of Human Rights”. The documents form the normative basis from which SGM rights were evaluated here, as an interconnected process across LAC countries. This methodological framework allowed us to conceptualize SGM policy as a social process, following a sociological dynamic of “increasing returns”, or positive feedback [53]. Furthermore, it integrated competing ideas, values and legislations from the different countries and sub-regions under analysis.

### Theoretical framework: path dependency in reactive sequences

The use of a path dependence methodology enables policy processes to be analyzed taking into account the significance of sequencing in policy development and policy changes. This framework was utilized to: (1) identify what aspects might define, review or change policies towards SGM from LAC countries; (2) create a reference framework to better understand the policy-making processes related to SGM rights; and (3) trace a possible interface between political changes, internal and external pressures and the (possible) adoption of new policies.

Our study considered the UDHR [50] as a critical juncture policy that influenced SGM rights protections internationally and within the LAC region, followed by ICCPR and ICESCR [51, 52]. Policy and time were key variables in our analysis, herein presented in diagrams that facilitate the visualization of their supposed interdependency in SGM rights [48]. We evaluated each selected policy to identify putative inputs, advantages, disadvantages and possible effects on other policies related to SGM rights in each country, sub-region and LAC.

### Data collection and search strategy

The first step, the identification stage of our search, was to extract relevant data from governmental policies and legislation that address SGM from all 33 LAC countries. Data was extracted from original legal documents published by each country. The search was not limited by language or publication year, as one major goal was to create a timeline of policies. The scope of SGM polices was narrowed to include two broad categories: legal protections addressing same-sex couples, and anti-discrimination laws.

Legal and policy documents retrieved from governmental websites or provided by governments were included if deemed eligible, whereas programs and strategies developed by non-governmental organizations were excluded. Identified laws were eligible regardless of publication date, and constitutions were included because of their fundamental role in each country’s legislations. Policy documents and governmental strategies were compared to and aligned in a timeline framework, organized by three sub-regions: the Caribbean (Antigua and Barbuda, Bahamas, Barbados, Cuba, Dominica, Dominican Republic, Grenada, Haiti, Jamaica, St. Kitts and Nevis, Saint Lucia, St. Vincent and the Grenadines, Trinidad and Tobago), Mesoamerica (Belize, Costa Rica, El Salvador, Guatemala, Honduras, Mexico, Nicaragua, and Panama) and South America (Argentina, Bolivia, Brazil, Chile, Colombia, Ecuador, Guyana, Paraguay, Peru, Suriname, Uruguay, Venezuela). Our study did not include overseas territories of the United Kingdom, the Netherlands, France or the USA.

### Data analysis

Our analysis included the following steps: (1) data extraction (date and description of legislation) for each country, (2) organization of legislation by sub-region (the Caribbean, Mesoamerica or South America), (3) aggrupation of legislations according to key domains under analysis, and (4) data analysis.

The domains analyzed addressed two broad categories: (1) legal protections addressing same-sex couples (decriminalization of same-sex acts among consenting adults, legal recognition of same-sex unions, same-sex marriage, adoption by same-sex couples), and (2) anti-discrimination laws (SGM allowed to serve openly in the military and anti-discrimination laws related to sexual orientation, gender identity and/or expression).

We conducted an analysis of policy path dependency in reactive sequences within each LAC sub-region. The strategy enables the identification of temporal patterns of policy-making, inter-relations between policies from the same country and possible temporal and political influences between countries and across a selected region. The strategy also allows for a better visualization of complex policy processes, such as those related to SGM rights. Policies that addresses highly vulnerable and stigmatized sub-groups tend to be deeply influenced by social, cultural and political changes, as well as national, regional and international scenarios [54]. This strategy was also adopted to (1) create a reference analysis framework, (2) identify changes in local legislations, following other countries new/updated laws, and (3) analyze the impact of different political scenarios (e.g. progressive vs. conservative) in SGM legislation in the region, following the reactive sequence theory.

The initial and seminal policy considered within our analysis was the UDHR [50], followed by ICCPR and ICESCR [51, 52] given that these documents were milestones that influenced international, regional and national policies addressing fundamental human rights.

Time and policy are two variables presented on a timeline to show their putative overlap, therefore facilitating the visualization and analysis of trends. This strategy allowed us to identify core policies as historical sequences and patterns, sometimes linear, sometimes not – therefore in synchronicity with the chosen theoretical framework. Current SGM-related policies were considered as a result of previous events, tracked using this framework.

## Results

**Fig. 1:**
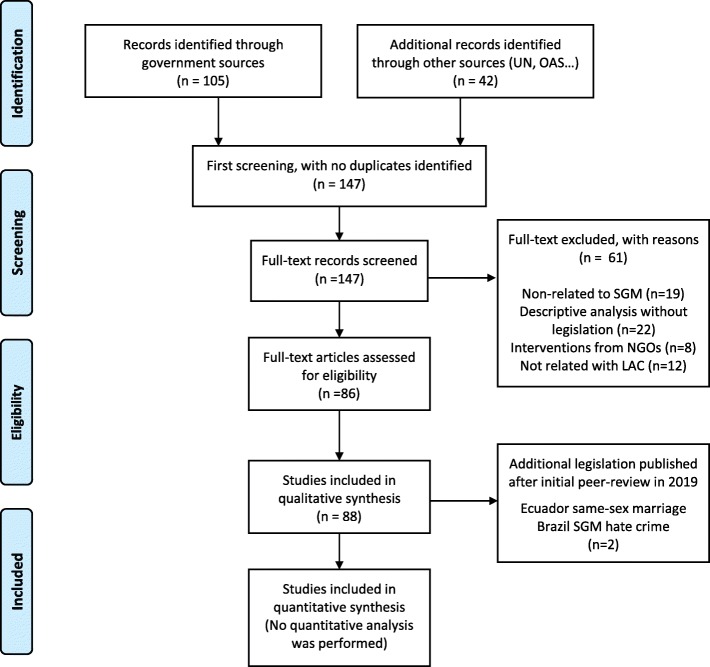
Overview of the search strategy according to the PRISMA flowchart

**Fig. 2:**
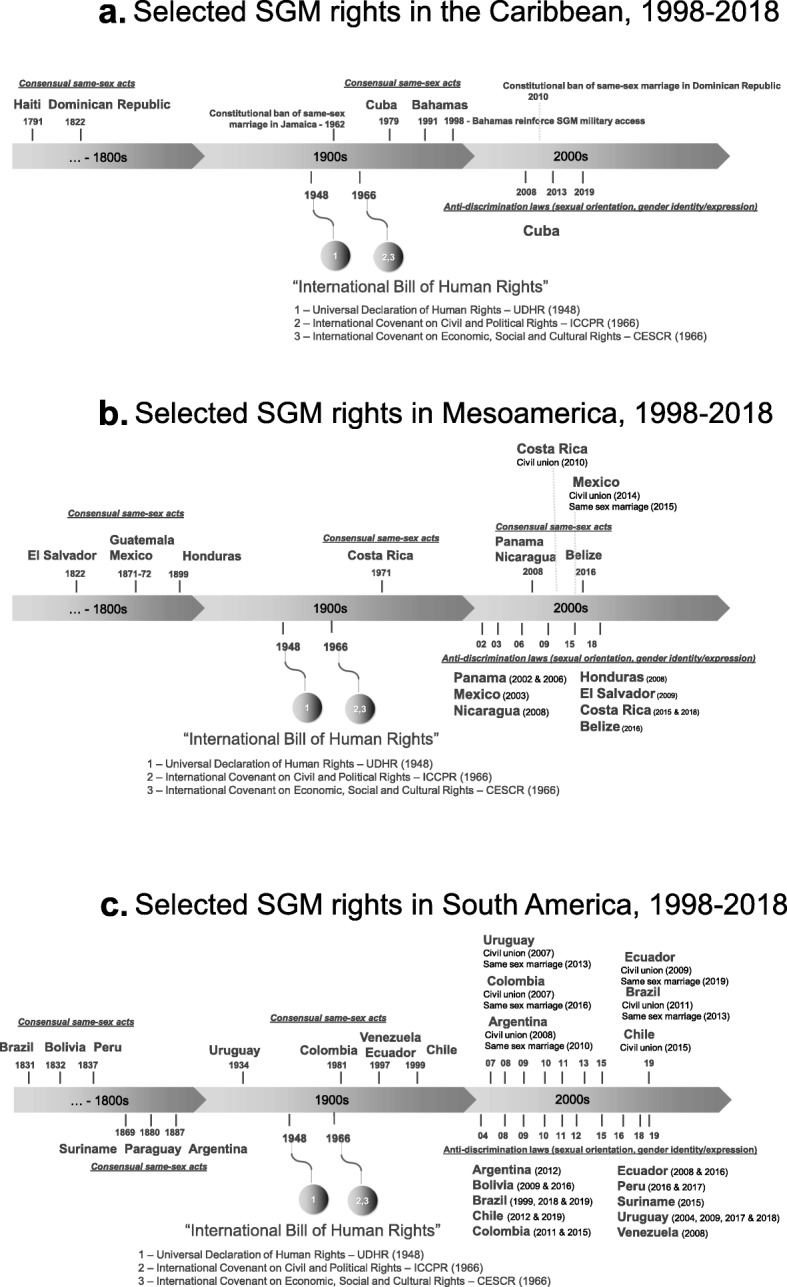
a Selected SGM rights in the Caribbean, 1998-2018. **b** Selected SGM rights in Mesoamerica, 1998-2018. **c** Selected SGM rights in South America, 1998-2018

We identified 105 documents through governmental sources and 42 additional reports from selected entities (Organization of American States, United Nations, Amnesty International, and Human Rights Watch). No duplicates were identified; therefore, we included 147 documents in the screening stage. During the screening, we excluded 61 records that did not mention our study population (SGM), did not describe any SGM-related legislation or were unrelated to the LAC region. The final number of policies initially included in our analysis was 86. After the manuscript initial peer-review, two new legislations were approved in 2019 (same-sex marriage in Ecuador and SGM-hate crime in Brazil). Therefore, we included 88 policies and international reports in our analysis. A full list of the policy documents analyzed in this paper is available (Additional file 1). Please see our PRISMA flow chart (Fig. 1) for the detailed search strategy stages and results. The countries were organized by sub-regions (Caribbean, Mesoamerica and South-America), to account for heterogeneity across LAC countries and to compare countries with more similar social and cultural characteristics. With these inputs, we created a timeline of policies related to SGM population for each country, organized by the three sub-regions under analysis (Fig. 2).

Table 1 summarizes the existing laws addressing SGM in each LAC country by sub-regions. All selected documents and reports are available at an Open Science Framework page (https://osf.io/4zq7j/files/?view_only=e139c918d77c4f14a120658edfd25804).

**Table 1:** Selected SGM rights in Latin America and the Caribbean, 1998–2018

Sub-region and Country	Legal protections addressing same-sex couples				SGM anti-discrimination laws		
	Consensual Same-Sex Sexual Acts	Recognition of same-sex legal union	Same-sex Marriage	Adoption by same-sex couples	SGM allowed to serve openly in military	Anti-Discrimination laws related to sexual orientation	Laws related to gender identity/expression
Caribbean							
Antigua & Barbuda	Illegal (only male, max penalty 15 years prison)	No	No	No	No	No	No
Bahamas	Legal since 1991	No	No	No	Yes (reinforced since 1998)	No	No
Barbados	Illegal (only male, max penalty for buggery life imprisonment)	No	No	No	No	No	No
Cuba	Legal since 1979	No	No	No	Since 1993	Yes Work-related anti-discrimination law (2013) New Constitution bans all anti SGM discrimination (2019)	Yes Since 2008 integral medical care (including gender-affirming surgery and hormone therapy) is provided by the government. Right to change legal gender and name since 2008, requiring gender-affirming surgery, medical certification and judicial procedures.
Dominica	Illegal (only male, max penalty 10 years in prison + psychiatric treatment	No	No	No	No	No	No
Dominican Republic	Legal since 1822	No	No (Constitutional ban since 2010)	No	No	No	No
Grenada	Illegal (only male, max penalty 10 years prison)	No	No	No	The country has no military	No	No
Haiti	Legal since 1791	No	No	No	The country has no military	No	No
Jamaica	Illegal (all genders^a^, max penalty 10 years prison + hard labor)	No	No (Constitutional ban since 1962)	No	No	No	No
St. Kitts and Nevis	Illegal (only male, max penalty 10 years prison + hard labor)	No	No	No	No	No	No
Saint Lucia	Illegal (only male, max penalty10 years in prison)	No	No	No	The country has no military	No	No
St. Vincent and the Grenadines	Illegal (all genders, max penalty 10 years prison)	No	No	No	The country has no military	No	No
Trinidad and Tobago	Legal since 2018	No	No	No	No	No	No
Mesoamerica							
Belize	Legal since 2016	No	No	No	No	Since 2016 bans all anti SGM discrimination	No
Costa Rica	Legal since 1971	Since 2014	No	No	The country has no military	Since 2015 bans all anti SGM discrimination	Since 2018 transgender persons can change their name and gender without conditions^b^
El Salvador	Legal since 1822	No	No	No	Since 1974	Since 2010 a decree bans anti SGM discrimination in public service	No
Guatemala	Legal since 1871	No	No	No	NA	In 1997 Code on Childhood & Youth bans discrimination based on sexual orientation against minors	Since 2016 TG can change legal name after judicial procedures, but not their legal gender
Honduras	Legal since 1899	No	No (Constitutional ban since 2005)	No	No	Since 2008 bans all anti SGM discrimination	No
Mexico	Legal since 1872	Since 2010 in all states and Mexico city	Since 2009 in Mexico city. Currently legal in Mexico city and 18/31 states	Since 2010 in Mexico city. Currently legal in 16/31 states and Mexico City	NA	Since 2003 bans all anti SGM discrimination (Federal Law to Prevent & Eliminate Discrimination) In 2013 Supreme Court ruled against hate speech towards SGM	TG can change legal name and gender in 6/31 states and Mexico City (2014) without conditions^b^ Mexican Supreme Court of Justice ruled that the right to self-determination of gender identity is a fundamental human right in 2019
Nicaragua	Legal since 2008	No (Constitutional ban since 2015)	No (Constitutional ban since 2015)	No (Constitutional ban since 2015)	NA	2008 – Labor rights & anti-discrimination	No
Panama	Legal since 2008	No	No	No	The country has no military	2002 – Anti-discrimination in public buildings/services	Since 2006 TG can change legal name and gender, but only after gender-affirming surgery. Since 2016 TG can change name without gender affirming surgery, but not gender.
South America							
Argentina	Legal since 1887	Since 2008	Since 2010	Since 2010	Since 2009	Since 1988 overall anti-discrimination law. No specific and national anti SGM discrimination	Since 2012 TG persons can change their name and gender without conditions^b^
Bolivia	Legal since 1832	No (Constitutional ban since 2009)	No (Constitutional ban since 2009)	No (Constitutional ban since 2009)	Since 2015	Since 2010 bans anti SGM discrimination (≈Law Anti-Racism)	Since 2016 TG persons can change their name and gender without conditions^b^
Brazil	Legal since 1831	Since 2011	Since 2013	Since 2010	Since 2015	Since 1999 - Ban ‘conversion therapy’ Since 2013 SGM Health Policy Since 2019 bans anti SGM discrimination (≈Law Anti-Racism)	Since 2018 TG persons can change their name and gender without conditions^b^
Chile	Legal since 1999	Since 2015	No	No	Since 2012	Since 2012 bans all anti SGM discrimination	Since 1974 TG can change legal name/gender. Since 2019 TG persons can change their name and gender without conditions^b^
Colombia	Legal since 1981	Since 2007	Since 2016	Since 2015	Since 1999	Since 2011	Since 2015 TG persons can change their name and gender without conditions^b^
Ecuador	Legal since 1997	Since 2009	Since 2019	No (Constitutional ban since 2009)	NA	Since 1998 Constitutional ban of discrimination based on sexual orientation Since 2008 bans all anti SGM discrimination Since 2014 - ban ‘conversion therapy’	Since 2016 TG persons can change their name and gender without conditions^b^ The government includes a permanent marker on documents to identify gender changes
Guyana	Illegal (male only, max penalty life prison)	No	No	No	Since 2012	No	No
Paraguay	Legal since 1880	No (Constitutional ban since 1992)	No (Constitutional ban since 1992)	No	Since 2010	No	No
Peru	Legal since 1836–1837	No	No	No	Since 2009	Since 2017 a decree bans all anti SGM discrimination	Since 2016 transgender persons can change their name and gender without gender-affirming surgeries. Judicial procedures required
Suriname	Legal since 1869	No	No	No	No	Since 2015	No
Uruguay	Legal since 1934	Since 2007	Since 2013	Since 2009	Since 2009	Since 2004 bans all anti SGM discrimination Since 2017 - ban ‘conversion therapy’	Since 2009 transgender persons can change their name and gender without gender-affirming surgeries. Since 2018 without judicial procedures.
Venezuela	Legal since 1800s	No	No	No	No	2008 - Supreme Court reinforces non-discrimination related to sexual orientation	No

^a^In Jamaica female same-sex encounters are not explicitly outlawed

^b^Without conditions: Name and gender change in official documents are allowed without the requirement of total or partial gender-affirming surgery, hormone therapies, medical/psychological treatment or evaluations. No legal or judicial procedures are required

### The Caribbean region

Discriminatory laws against SGM people are particularly common in the Caribbean region. The majority of Caribbean countries have versions of “buggery” and gross indecency laws, relics of British colonialism, that prohibit same-sex conduct between consenting adults. Among the countries evaluated (*n* = 13), the majority has laws that, when violated, include 10 years to life imprisonment, fines and/or hard work: Antigua and Barbuda, Barbados, Dominica, Grenada, Jamaica, St. Kitts and Nevis, St. Lucia, and St. Vincent and the Grenadines (Table 1). Although these laws are vaguely worded and rarely enforced in criminal prosecutions, the legislation contributes to widespread discrimination, violence, stigma and prejudice against SGM in the region [55].

Section 9 of the Sexual Offences Act of Barbados criminalizes the act of “buggery”, defined by the Barbadian courts as “anal sex between men or between a man and a woman”. Section 12 criminalizes “serious indecency,” which is defined as any act by anyone “involving the use of the genital organs for the purpose of arousing or gratifying sexual desire.” These acts are criminalized notwithstanding the consent of the participants. The maximum penalty for buggery is life imprisonment; the maximum penalty for an act of serious indecency (involving a partner above the age of 16) is 10 years in prison (Table 1).

In Haiti, there is no law criminalizing consensual same-sex sexual acts, but Article 227 of its criminal code prohibits vagrancy, with a specific mention in the code to transgender people. In 2017, the Haitian Senate passed a Bill listing homosexuality, alongside child pornography and incest, as reasons to deny a citizen its *‘Certificat de Bonne Vie et Moeurs’*, a document required by employers and universities. In the same year, another Bill was passed to ban gay marriage and any public advocacy of SGM rights [48]. Only Cuba and Bahamas allow SGM to serve openly in the military. Cuba is the single country in the Caribbean with anti-discrimination laws related to sexual orientation (2014) and gender identity/expression (2008). Since 2008, Cuba’s government provides integral medical care (including gender-affirming surgeries and hormonal treatment) through the National Health System. Transgender people can change their name and gender in legal documents, but need to submit the petition to a local tribunal, including a medical certification that the petitioner has undergone gender-affirming surgery (Table 1).

The key international documents that guided our analysis (UDHR [1948], ICCPR [1966] and ICESCR [1966]) do not seem to have influenced policies targeting SGM in most countries in the Caribbean. After the publication of the ‘International Bill of Human Rights’, only Cuba implemented a broad range of new policies allowing consensual same-sex acts (1979), transgender individuals to change their legal name and gender (2008), work-related SGM anti-discrimination laws (2014), and overall SGM anti-discrimination laws (2019). Besides Cuba, the Bahamas also implemented a new legislation allowing consensual same-sex acts (1991) and reinforced the right of SGM to serve openly in the military (1998). In contrast, after the publication of the ‘International Bill of Human Rights’, two countries adopted a constitutional ban against same-sex marriage: Jamaica (1962) and the Dominican Republic (2010).

SGM prosecutions are not common in the region, but the overall lack of legal protection contributes to an environment that condones discrimination, stigmatization, and violence towards SGM. These laws (or the lack of legislation against discrimination) have frequently been utilized to justify arbitrary detention, police abuse, extortion and torture. SGM who are incarcerated or otherwise implicated in the justice system are particular targets for sexualized violence and administrative punishment while in custody.

Our analysis identified that discriminatory legislations (Table 1) negatively influence SGM rights the Caribbean, making them ready victims of discrimination, violence, and other human rights violations. The majority of Caribbean countries do not seem to be influenced by the ‘International Bill of Human Rights’ and its core principles to promote and protect human rights of individuals and more vulnerable groups, such as SGM (Fig. 2a).

To some extent, Cuba remains a single exception in this scenario. As of August 2019, the country signed in 2008 the ICCPR and ICESCR, but did not ratify these treaties. In spite of the identified progress in the field of SGM-rights, the Amnesty International and Human Rights Watch have reported several human rights violations in Cuba. The state tight controls freedom of expression, and frequently employ arbitrary detention to harass and intimidate critics, independent activists, political opponents and human rights defenders who attempt to document abuses – including SGM citzens. In 2019, the government canceled the annual LGBT Pride Parade and arrested SGM activists who organized an unauthorized march.

### Mesoamerica region

We included in this sub-region seven countries from Central America (Belize, Costa Rica, El Salvador, Guatemala, Honduras, Nicaragua and Panama) and Mexico. Same-sex sexual acts among consenting adults has been legal in four countries since the 1800’s (El Salvador, Guatemala, Honduras and Mexico). A few years after the publication of the ‘International Bill of Human Rights’, same-sex sexual acts among consenting adults became legal in Costa Rica (1971). However, the same legislation was only adopted in Nicaragua and Panama in 2008, followed by Belize in 2016 (Fig. 2b).

In the vast majority of countries in Mesoamerica, same-sex couples do not have equal marriage rights and are not allowed to adopt as a couple. In January 2015, the Supreme Court of Mexico handed down a landmark decision in which the legal definition of marriage was changed to encompass same-sex couples. After this decision, the law restricting marriage to a man and a woman was considered unconstitutional. However, in several states of Mexico, same-sex couples still have to request an injunction (“Amparo”) from a judge to marry, a process not required for opposite-sex couples. In Costa Rica, same-sex legal unions are recognized (2014). On the opposite end of the spectrum, although same-sex sexual activity among consenting adults is legal in Honduras and Nicaragua, both countries have constitutional bans against same-sex marriage. In Honduras, same-sex marriages, de facto unions and adoption by same-sex couples were constitutionally banned in 2005. In Nicaragua, after the adoption of a new Family Code (2015), marriage, civil union or adoption by same sex couples was prohibited. In Mexico, only 16 states (of 31) and Mexico City allow adoption by same-sex couples, and until the end of this review (August 2019) there was no national legislation addressing marriage equality (Table 1). In contrast, in April 2017, lawmakers from Guatemala presented a legislative proposal to explicitly prohibit same-sex marriage and restrain public schools from teaching about sexual diversity and gender identity – the proposal was supported by over 30,000 signatures.

Four countries implemented some degree of anti-SGM discrimination laws, including labor rights legislations in Nicaragua (2008), anti-discrimination laws in public buildings in Panama (2002) and El Salvador (2010), and legislation protecting SGM youth in Guatemala (1997). Four countries ban all discrimination based on sexual orientation and gender identity: Mexico (2003), Honduras (2008), Costa Rica (2015) and Belize (2016).

Only Costa Rica allows transgender people to change their name and gender on documents without surgeries, medical/psychological evaluations or judicial permission. Since 2006, Panama allows transgender persons to change their legal name and gender, but only after gender affirming surgery. Since 2016, transgender people can change their name without needing any surgery in Panama, but not their gender. In Guatemala, transgender persons can change their legal name after judicial permission, but not their legal gender (2016). In Mexico, there is no national legislation addressing the needs and specificities of transgender people. Since 2015, transgender people can change their legal name and gender in Mexico City without the need of psychological/medical evaluations or gender-affirming surgery. Six states (of 31) adopted similar legislations until the end of this review (August 2019). In May 2019, the Mexican Supreme Court of Justice decided that the right to self-determination of gender identity is a fundamental human right; therefore, a new scenario might be implemented towards transgender rights in the entire country. (Table 1).

In May 2016, the government of Costa Rica questioned the Inter-American Court of Human Rights, of the Organization of American States (IACHR/OAS) about their obligations related to transgender citizens who requested to change their names and gender in official documents. They also inquired if the country should develop a specific legislation to recognize the economic rights of same-sex couples [56]. In 2018, the IACHR/OAS affirmed, through Advisory Opinion 24/17, that all State Members should assure that same-sex couples have access to civil marriage and all related rights. The document also requires State Members to offer a fast, easy and cost-free process to allow transgender people legally change their name and gender in accordance with their self-perceived gender identity [56]. Following IACHR/OAS Advisory Opinion 24/17, in 2018 Costa Rica implemented a new legislation that allows transgender person to change their legal name and gender without the need of medical/psychological evaluation, judicial permission or gender-affirming surgery.

However, in Mesoamerica systemic discriminatory legislations seems to exacerbate the discrimination towards SGM, within a social and cultural context of strong sexism, gender stereotypes and high rates of violence. This scenario is driving hundreds of SGM from Mesoamerica to leave their home countries, usually looking for refuge in Mexico or in the United States [57]. According to the Office of the United Nations High Commissioner for Refugees (UNHCR), nearly 90% of SGM asylum seekers and refugees from Central America report experiences of sexual and gender-based violence in their home countries [58].

### South America region

Same-sex sexual acts among consenting adults has been legal in six countries of South America since 1800’s (Argentina, Brazil, Bolivia, Paraguay, Peru, and Suriname). Four countries legalized same-sex consensual acts during the 1990’s: Chile, Colombia, Ecuador and Uruguay. In Venezuela same-sex sexual acts was never illegal, but a law related to ‘Vagrants and Thugs’ (*‘Ley de vagos y maleantes’*) was occasionally applied to SGM engaged in sex work. The Supreme Court of Justice declared this law unconstitutional in 1997. Same sex acts between two men is illegal in Guyana and could lead to imprisonment for life (Fig. 2c).

Six countries recognize same sex legal unions: Argentina, Brazil, Chile, Colombia, Ecuador and Uruguay. Argentina, Colombia, Brazil and Uruguay also recognize same-sex marriage nationally and allow same-sex couples to adopt. Following IACHR/OAS Advisory Opinion 24/17 [56], in July 2019 same-sex marriage became legal in Ecuador, but the country does not allow same-sex couples to adopt. As of August 2019, only two countries in the region have a constitutional ban against same-sex marriage: Paraguay (1992) and Bolivia (2009).

Guyana is the only country in South America, and the only country in the Americas outside the Caribbean, where homosexual acts (as well as heterosexual anal and oral sex) are illegal. Guyana does not recognize same-sex legal unions; neither allow same-sex couples to adopt. In the country, transgender people cannot change their legal name and gender, and there is no SGM anti-discrimination legislation. In November 2018, Guyana’s final court of appeal ruled that a law against cross-dressing violates the constitution of Guyana, voiding it. Guyana, Paraguay, Suriname and Venezuela are the countries where SGM people have fewer rights in the region (Table 1).

Transgender individuals can change their legal name and gender in Argentina, Bolivia, Brazil, Chile, Colombia, Ecuador and Uruguay without the requirement of surgeries, medical/psychological evaluations or legal procedures. In Peru, transgender people can change their name and gender, but they need to apply before a judge - no medical/psychological evaluations or surgeries are required. Those legislations were implemented after the ‘International Bill of Human Rights’, mostly during the last two decades (Fig. 2c).

Overall, legislation to protect SGM rights in South America have undergone fundamental and positive transformations during the last decade. The majority of countries in South America have legal protections against discrimination towards SGM people and consider hate crimes based on sexual orientation and/or gender identity as an aggravating circumstance. Eight countries (of 12) allow transgender people to change their name and gender on legal documents without surgeries or medical/psychological evaluations, just one requiring judicial permission **(**Table 1**)**.

Despite the expanding federal laws to protect SGM, the region registers one of the highest rate of violence and hate crimes against SGM people. Whichever the legal framework, and regardless of whether it includes specific hate crime or hate speech laws addressing SGM people, each country need to ensure that criminal legislation relating to SGM discrimination and violence is diligently applied. The existence of laws addressing SGM rights violation is important, but if available legislation is not effectively enforced a mutually reinforcing cycle of lack of reporting and low conviction rates continues. The region countries need to created effective mechanisms to enforce these laws. In addition, the majority of countries do not classify crimes based on sexual orientation and/or gender identity as hate crimes, with more severe penalties. The lack of proper law implementation might be a key factor influencing high rates of violence against SGM in the region. Brazil, for instance, has the highest rate of hate crimes against SGM in the world – crimes frequently unprosecuted and unpunished. Only in June 2019, the Brazilian Supreme Court decided to consider discrimination on the grounds of sexual orientation or gender identity equivalent to racism, making those crimes punishable under the country’s more severe anti-racism law.

In recent years, highly conservative groups have also increased their political influence in the South America region. In January 2019, Brazil’s far-right president, Jair Bolsonaro, was sworn into office. Under Mr. Bolsonaro administration, an ultra-conservative agenda is being implemented, deeply decreasing the country progressive support towards diversity and human rights [59, 60]. The government authorized severe cuts in federal funding for education, health, research and social support strategies, directly affecting SGM people and other vulnerable minorities [42].

In Venezuela, Nicolás Maduro was first elected president in 2013, and re-elected in a controversial election in 2018. Until mid-2019, over 4 million people have left Venezuela to escape violence, insecurity, life threats, lack of food, medicine and essential services – the percent of those who self-identify as SGM is unknown [61]. After a decade of leftist government, around mid-2010 a right-wing political phenomenon emerged in South America. Currently the majority of countries in the region are controlled either by center-right (e.G. *Argentina*), right wing leaders (e.g. Brazil) or by repressive dictators (Venezuela). The near future of the region is unknown, but if this highly conservative political agenda influence human rights legislation currently available, setbacks in SGM legal protections are likely.

## Discussion

Recently the IACHR/OAS Advisory Opinion 24/17 stated that all signatory member States of the of the American Convention on Human Rights (‘Pact of San Jose’, signed in 1969) should assure that same-sex couples have access to civil marriage and all related rights. The Advisory Opinion also requested State Members to assure that adequate and efficient gender identity legislations are available to their transgender citizens [56].

In terms of marriage equality, this IACHR Advisory Opinion 24/17 could be an important step forward to promote SGM rights in the LAC region. However, among 20 states from LAC that recognize the IACHR and the Court mandatory jurisdiction, 14 have not yet legalized same-sex marriage: Barbados, Bolivia, Chile, Costa Rica, Dominican Republic, El Salvador, Guatemala, Haiti, Honduras, Nicaragua, Panama, Paraguay, Peru, and Suriname [56]. Trinidad and Tobago and Venezuela had both ratified the Court’s jurisdiction, but withdrew its ratification when they denounced the American Convention in 1998 and 2012, respectively – both countries do not recognize same-sex marriage. Grenada, Dominica and Jamaica ratified the ‘Pact of San Jose’, but do not recognize the IACHR mandatory jurisdiction – those countries likewise do not recognize same-sex marriage.

IACHR’s Advisory Opinion 24/17 also addressed legal gender recognition, recommending all signatory countries to adopt a simplified process allowing anyone to change their name and affirm their self-perceived gender identity in public records and documents. According to IACHR, the process should be confidential, free, and not require surgery or hormone treatment [56]. However, until August 2019, transgender people are unable to change their gender in public records and legal documents in several LAC countries – mostly in the Caribbean and Mesoamerica. Other countries allow those changes only through a complex, costly and time-consuming process involving judicial permission and court appearances (as in Guatemala), or only after gender-affirming surgery (as in Panama and Cuba). Other countries, like Ecuador, allow transgender citizens to change their name and gender, but include a permanent marker on the person’s documents – a stigmatizing policy.

On the opposite side of the spectrum, the majority of countries from South America allow their transgender citizens to change name and gender in legal documents with a fast, easy and inexpensive manner. On February 3rd, 2018, the Brazilian Supreme Court authorized gender modification on civil documents without the need of any medical evaluation or gender-affirming surgery. The Brazilian Superior Electoral Court allowed transgender candidates to use their preferred name and pronouns during the 2018 elections. In June 2019, the Brazilian Supreme Court made homophobia and transphobia crimes similar to racism, a hate crime [62].

In most countries where transgender people are refused legal recognition of their gender identity, this might lead to further human rights violations, impacting their access to education, employment, healthcare, social security, and legal protection [29, 30]. Many countries that do permit the modification of gender markers on identity documents impose abusive requirements, such as forced or otherwise involuntary surgery, medical diagnosis, long, costly and complicated judicial procedures [31, 32]. The translation of IACHR Advisory Opinion 24/17 into reality will require multidisciplinary work and strong political will in the LAC region. However, in the past few years conservative movements have resulted in a strong backlash against SGM rights across the Americas. In 2016, a conservative movement contributed to blockage of a constitutional Bill on marriage equality in Mexico. In 2017, the conservative parties from Peru derailed a proposed SGM-inclusive education curriculum. Recently, people have rallied in Paraguay and Ecuador, claiming a need to defend the “traditional family” from so-called “gender ideology” [57, 63, 64]. Right wing and ultra conservative presidents have been elected in Argentina (2015), Chile (2017), and Brazil (2018).

While studying the uneven evolution of SGM rights in the LAC region, it is possible to state that the existence of pro-SGM movements and progressive legislations have been important but not sufficient to advance SGM rights. The expansion of SGM rights and efforts to advance those rights have produced a persistent confrontation between right-based social movements, the State and conservative/religious organizations. In the region, religiosity and faith-based groups continue to be strong veto players and frequently influence the implementation of more inclusive SGM-rights legislations [65].

Our analysis demonstrated that countries located in the Central America and the Caribbean provide less constitutional rights to their SGM citizens. SGM related social movements are recent within this region and started in the 1990’s to fight the AIDS epidemic. Until nowadays SGM-rights efforts in the region face strong opposition from governments, religious organizations and the society at large [38–40].

### Policy challenges, opportunities and the way forward

This review discussed the transformation of SGM rights in Latin America and the Caribbean. The different scenarios provide important examples of how social movements, institutions and governments can (and should) work together to improve SGM rights. Social movements within the LAC region have made impressive advances in the legal environment of many countries, despite the prevalence of adverse cultural attitudes, conservative norms and prejudice.

While there has been an undeniable progress in many countries, stagnation on the legal status of SGM people is also visible in the LAC region. In some countries (e.g., Argentina, Uruguay, Brazil) and in some cities (e.g., Mexico City), the legal status of SGM rights is ahead of some of the most advanced and democratic nations of the world – with same-sex marriage or civil unions, anti-discrimination and hate crime laws, and progressive legislations addressing transgender rights. In other countries (mostly in the Caribbean and Central America), the legal status of SGM rights remains underdeveloped. Sometimes this unevenness is perceived in the same country. Brazil, for instance, provides extensive legal protections to SGM, but has also one of the highest rates of SGM violence and murders worldwide [59, 60].

Reasons for this disparity are complex and represent a clear indication that progressive legislation is not sufficient to advance SGM quality of life. In a social, cultural and political scenario of entrenched prejudice, discrimination and rampant violence, SGM-related legislation is frequently not enforced or even observed. Few Latin American employers have proactive policies towards SGM employees. Marriage continues to be defined, constitutionally in some cases, as the union between a man and a woman. Transgender people are frequently victims of harassment, discrimination, sexual and physical violence (committed by police officers, other State officials and non-State actors). Although the legislation exists (in some countries), the cultural and social environment is unwelcoming and constantly dangerous for SGM in the LAC region.

## Conclusion

International courts are not be able to protect the rights of SGM people due to region and country-specific legislations. Political, cultural and societal pressures constantly influence how (and if) legislation and national policies underpin a rights based approach or not. Unfortunately, just a few national and subnational courts in the LAC region respect all signed international laws and decrees, as clearly presented here. However, in countries with an independent and impartial judiciary, where governments understand and respect human rights standards, those international laws should contribute and inform a necessary pathway to change.

Recent advances show good prospects, but the processes of constructing non-discriminatory public policies towards SGM face several barriers in each country. Cultural norms and the overall political environment highly influence SGM rights in the region and worldwide. We conclude that SGM rights in the LAC region need to be improved. Governments and the civil society should work together, in order to tackle the unacceptable levels of violence and human rights violations towards SGM in the region. It is also pivotal to increase this population access to health, education, social support, legal protection and justice.

The policy synthesis provided here could guide LAC policymakers, political and government leaders, advocates, lawyers, policy makers and activists to identify local and regional gaps in SGM-related rights. Our study can also underscore the need to advocate for better legislations that respect the rights and dignity of all. The Universal Declaration of Human Rights [50] states in Article 2: “Everyone is entitled to all the rights and freedoms set forth in this Declaration, without distinction of any kind.” According to the United Nations [66], failure by State authorities to investigate and punish SGM-related discrimination and violence “is a breach of the State’s obligation to protect everyone’s right to life, liberty and security of person, as guaranteed by article 3 of the UDHR and articles 6 and 9 of the ICCPR”.

Regardless, in 2019 a substantial percentage of signatory Member States fail to protect the human rights of their SGM citizens. LAC countries have achieved several successes in this field, and have the potential to foster SGM rights in the next years. The question is whether local governments and lawmakers will actually commit their efforts to protect human rights and decrease the unacceptable abuses towards SGM identified in the Latin America and the Caribbean.

## Supplementary information

**Additional file 1.** List of Selected documents.

## Data Availability

All selected documents are available at an Open Science Framework: https://osf.io/4zq7j/?view_only=e139c918d77c4f14a120658edfd25804

## Abbreviations

IACHR: Inter-American Court of Human Rights
LAC: Latin America and the Caribbean
LGBTI: Lesbian, Gay, Bisexual, Transgender
OAS: Organization of American States
UNHCR: Office of the United Nations High Commissioner for Refugees

## References

[1] CSDH (2008). Closing the gap in a generation: health equity through action on the social determinants of health. Final report of the commission on social determinants of health.

[2] Marmot M, Friel S, Bell R, Houweling TA, Taylor S (2008). Commission on Social Determinants of Health. Closing the gap in a generation: Health equity through action on the social determinants of health. Lancet.

[3] MARMOT M (2005). Social determinants of health inequalities. The Lancet.

[4] Institute of Medicine (2011). The health of lesbian, gay, bisexual, and transgender people: building a foundation for better understanding.

[5] Case P, Austin SB, Hunter DJ, Manson JE, Malspeis S, Willett WC, Spiegelman D (2004). Sexual orientation, health risk factors, and physical functioning in the Nurses' health study II. J Women's Health (Larchmt).

[6] Caceres BA, Makarem N, Hickey KT, Hughes TL (2019). Cardiovascular disease disparities in sexual minority adults: an examination of the behavioral risk factor surveillance system (2014-2016). Am J Health Promot.

[7] Mojola SA, Everett B (2012). STD and HIV risk factors among U.S. young adults: variations by gender, race, ethnicity and sexual orientation. Perspect Sex Reprod Health.

[8] Boyd CJ, Veliz PT, Stephenson R, Hughes TL, McCabe SE (2019). Severity of alcohol, tobacco, and drug use disorders among sexual minority individuals and their "not sure" Counterparts.LGBT. Health..

[9] White Hughto JM, Pachankis JE, Willie TC, Reisner SL (2017). Victimization and depressive symptomology in transgender adults: the mediating role of avoidant coping. J Couns Psychol.

[10] Davies RD, Kessel B (2017). Gender minority stress, depression, and anxiety in a transgender high school student. Am J Psychiatry.

[11] Veale JF, Watson RJ, Peter T, Saewyc EM (2017). Mental health disparities among Canadian transgender youth. J Adolesc Health.

[12] Nguyen KH, Trivedi AN, Shireman TI (2018). Lesbian, gay, and bisexual adults report continued problems affording care despite coverage gains. Health Aff (Millwood).

[13] Jennings L, Barcelos C, McWilliams C, Malecki K (2019). Inequities in lesbian, gay, bisexual, and transgender (LGBT) health and health care access and utilization in Wisconsin. Prev Med Rep.

[14] Conron KJ, Mimiaga MJ, Landers SJ (2010). A population-based study of sexual orientation identity and gender differences in adult health. Am J Public Health.

[15] Valdiserri RO, Holtgrave DR, Poteat TC, Beyrer C (2019). Unraveling health disparities among sexual and gender minorities: a commentary on the persistent impact of stigma. J Homosex.

[16] Cruz TM (2014). Assessing access to care for transgender and gender nonconforming people: a consideration of diversity in combating discrimination. Soc Sci Med.

[17] Charlton BM, Gordon AR, Reisner SL, Sarda V, Samnaliev M, Austin SB (2018). Sexual orientation-related disparities in employment, health insurance, healthcare access and health-related quality of life: a cohort study of US male and female adolescents and young adults. BMJ Open.

[18] Goldhammer H, Maston ED, Kissock LA, Davis JA, Keuroghlian AS (2018). National Findings from an LGBT Healthcare Organizational Needs Assessment. LGBT Health.

[19] Meyer IH (1995). Minority stress and mental health in gay men. J Health Soc Behav.

[20] Hendricks ML, Testa RJ (2012). A conceptual framework for clinical work with transgender and gender nonconforming clients: an adaptation of the minority stress model. Prof Psychol Res Pract.

[21] Bockting WO, Miner MH, Swinburne Romine RE, Hamilton A (2013). Coleman E stigma, mental health, and resilience in an online sample of the US transgender population. Am J Public Health.

[22] Meyer IH (2003). Prejudice, social stress, and mental health in lesbian, gay, and bisexual populations: conceptual issues and research evidence. Psychol Bull.

[23] Blondeel K, Say L, Chou D, Toskin I, Khosla R, Scolaro E, Temmerman M (2016). Evidence and knowledge gaps on the disease burden in sexual and gender minorities: a review of systematic reviews. Int J Equity Health.

[24] The Lancet Public Health (2019). LGBT: the vital fight for the right to health. Lancet Public Health.

[25] Buffie WC (2011). Public health implications of same-sex marriage. Am J Public Health.

[26] Herdt G, Kertzner R (2006). I do, but I can't: the impact of marriage denial on the mental health and sexual citizenship of lesbians and gay men in the United States. Sexuality Research and Social Policy.

[27] Tatum AK (2017). The interaction of same-sex marriage access with sexual minority identity on mental health and subjective wellbeing. J Homosex.

[28] Riggle ED, Rostosky SS, Horne SG (2010). Psychological distress, well-being, and legal recognition in same-sex couple relationships. J Fam Psychol.

[29] Du Bois SN, Yoder W, Guy AA, Manser K, Ramos S (2018). Examining associations between state-level transgender policies and transgender health. Transgend Health.

[30] Gruskin Sofia, Everhart Avery, Olivia Diana Feliz, Baral Stefan, Reisner Sari L, Kismödi Eszter, Cruz David, Klemmer Cary, Reich Michael R, Ferguson Laura (2018). “In transition: ensuring the sexual and reproductive health and rights of transgender populations.” A roundtable discussion. Reproductive Health Matters.

[31] Winter S, Diamond M, Green J, Karasic D, Reed T, Whittle S, Wylie K (2016). Transgender people: health at the margins of society. Lancet..

[32] Divan V, Cortez C, Smelyanskaya M, Keatley J (2016). Transgender social inclusion and equality: a pivotal path to development. J Int AIDS Soc.

[33] Reisner SL, Hughto JM, Dunham EE (2015). Legal protections in public accommodations settings: a critical public health issue for transgender and gender-nonconforming people. Milbank Q.

[34] Stroumsa D (2014). The state of transgender health care: policy, law, and medical frameworks. Am J Public Health.

[35] Paula CEA, Silva APD, Bittar CML (2017). Legislative vulnerability of minority groups. Cien Saude Colet.

[36] Cahill SR, Makadon HJ (2017). If they Don't count us, we Don't count: trump administration rolls Back sexual orientation and gender identity data collection. LGBT Health.

[37] Gonzales G, McKay T (2017). What an emerging trump administration means for lesbian, gay, bisexual, and transgender health. Health Equity.

[38] Corrales J, Pecheny M. In: Charles J, Conaghan CM, editors. The politics of sexuality in Latin America: a reader on lesbian, Gay, Bisexual, and Transgender Rights. Pitt Latin American Series: University of Pittsburgh Press; 2010.

[39] Diez J (2011). Argentina: a queer tangle between the lesbian and gay movement and the state. In Trembaly, Manon & Paternotte, David, & Johnson, Carol (Eds.), the lesbian and gay movement and the state: comparative insights into a transformed relationship (pp. 13–25).

[40] Encarnacion O (2011). Latin America’s gay rights revolution. J Democr.

[41] Paim J, Travassos C, Almeida C, Bahia L, Macinko J (2011). The Brazilian health system: history, advances, and challenges. Lancet..

[42] Castro MC, Massuda A, Almeida G, Menezes-Filho NA, Andrade MV, de Souza Noronha KVM, Rocha R, Macinko J, Hone T, Tasca R, Giovanella L, Malik AM, Werneck H, Fachini LA, Atun R (2019). Brazil's unified health system: the first 30 years and prospects for the future. Lancet..

[43] Alencar Albuquerque G, de Lima GC, da Silva QG, Alves MJ, Belém JM, dos Santos Figueiredo FW, da Silva PL, Do Nascimento VB, da Silva Maciel É, Valenti VE, de Abreu LC, Adami F (2016). access to health services by lesbian, gay, bisexual, and transgender persons: systematic literature review. BMC Int Health Hum Rights.

[44] Drescher J, Schwartz A, Casoy F, McIntosh CA, Hurley B, Ashley K, Barber M, Goldenberg D, Herbert SE, Lothwell LE, Mattson MR, McAfee SG, Pula J, Rosario V, Tompkins DA (2016). The growing regulation of conversion therapy. J Med Regul.

[45] Strickler J (2017). Variation in Latin American LGBT rights.

[46] Díez J (2015). The politics of same-sex marriage in Latin America: Argentina.

[47] Encarnación O. Out in the periphery: Latin America’s gay rights revolution: Oxford University Press; 2016.

[48] Mahoney J (2000). Path dependence in historical sociology. Theory Soc.

[49] Pierson P (2000). Increasing Returns, Path Dependence, and the Study of Politics. Am Polit Sci Rev.

[50] United Nations (1948) Universal Declaration of Human Rights. New York, NY, USA: United Nations. Available at: https://www.ohchr.org/EN/UDHR/Documents/UDHR_Translations/eng.pdf Accessed in May 14, 2019.

[51] International Covenant on Civil and Political Rights – ICCPR. 1966. Available at: https://www.ohchr.org/en/professionalinterest/pages/ccpr.aspx Accessed May 14, 2019.

[52] International Covenant on Economic, Social and Cultural Rights – ICESCR. 1966. Available at: https://www.ohchr.org/en/professionalinterest/pages/cescr.aspx Accessed May 14, 2019.

[53] Pierson P (2000). Not just what, but when: timing and sequence in political processes. Studies in American Political Development.

[54] Bernier NF, Clavier C (2011). Public health policy research: making the case for a political science approach. Health Promot Int.

[55] Human Rights Watch. 2018. “I Have to Leave to Be Me”: Discriminatory Laws against LGBT People in the Eastern Caribbean. Available at: https://www.hrw.org/report/2018/03/21/i-have-leave-be-me/discriminatory-laws-against-lgbt-people-eastern-caribbean\.

[56] Inter-American court of human rights (IACHR). Advisory opinion OC-24/17 of November 24, 2017. Series A No 24. Available at http://www.corteidh.or.cr/docs/opiniones/seriea_24_esp.pdf

[57] Amnesty International. 'No safe place': Salvadorans, Guatemalans and Hondurans seeking asylum in Mexico based on their sexual orientation and/or gender identity Available at: https://www.amnesty.org/download/Documents/AMR0172582017ENGLISH.PDF

[58] Office of the United Nations High Commissioner for Human Rights (OHCHR). UN Human Rights Report: 2017. Available at: http://www2.ohchr.org/english/OHCHRreport2017/allegati/Downloads/1_Whole_Report_2017.pdf

[59] Malta M, Silva AB, LeGrand S, Whetten K, Wells S (2019). HIV/AIDS, human rights, and transgender people in Latin America. Lancet Public Health.

[60] Montenegro L, Velasque L, LeGrand S (2019). Whetten K, de Mattos Russo Rafael R, Malta M.

[61] United Nations High Commissioner for Refugees (UNHCR). “Venezuela situation”. Available at: https://www.unhcr.org/venezuela-emergency.html

[62] Human Rights Watch Country Profiles: Sexual Orientation and Gender Identity, 2019. Available at: https://www.hrw.org/video-photos/interactive/2019/02/28/sexual-orientation-gender-identity-country-profiles

[63] Viteri MA & Ocampo G. Sexual politics in Ecuador in the 2000’s: a bird’s eye view. Sexuality policy Watch, 2017. Available at: http://sxpolitics.org/sexual-politics-in-ecuador-in-the-2000s-a-birds-eye-view/17140#_ftn27

[64] Wilkinson A (2013). Sin sanidad, no hay santidad: las prácticas reparativas en Ecuador.

[65] Corrales J (2015). The politics of LGBT rights in Latin America and the Caribbean: research agendas. European Review of Latin American and Caribbean Studies.

[66] United Nations (2016). Living Free & Equal: what states are doing to tackle violence and discrimination against lesbian, gay, bisexual, transgender and intersex people.

