# Supplementary material for: Sexual and gender minorities rights in Latin America and the Caribbean: a multi-country evaluation
Source: BMC Int Health Hum Rights. 2019 Nov 6;19:31. doi: 10.1186/s12914-019-0217-3 (PMC6836409; doi:10.1186/s12914-019-0217-3)
Supplement: Supplementary file 1 — Additional file 1. List of Selected documents. [file 12914_2019_217_MOESM1_ESM.pdf]

Supplement 1: List of Selected documents

Documents and legislations from LAC countries (File name)	Description
Antigua_and_Barbuda_Constitution	Constitution of Antigua and Barbuda
Antigua_and_Barbuda_Criminal_Law_Amendment	Antigua and Barbuda Criminal Law Amendment Act
Antigua_and_Barbuda_The_Sexual_Offences_Act	Antigua and Barbuda act that replaces laws related to sexual crimes
Argentina_Civil_Union	Legislation that authorizes same-sex marriage in Argentina
Argentina_Constitution	Constitution of Argentina
Argentina_SGM_Rights	List of laws and rights related to SGM from Argentina
Bahamas_Act_to_Amend_the_Sexual_Offences_and_Domestic_Violence_Act_and_for_Incidental_Matters	Bahamas – amendment of sexual offences laws
Bahamas_Constitution_of_1973	Constitution of Bahamas
Bahamas_Sexual_Offences_and_Domestic_Violence_Act	Bahamas – legislation related to sexual offenses and domestic violence
Barbados_Constitution	Constitution of Barbados
Belize_Constitution	Constitution of Belize
Belize_Supreme_Court_Decision	Belize review of buggery law
Belize_Supreme_Court_Process	Belize review of same-sex criminalization law
Bolivia_Constitution	Constitution of Bolivia
Bolivia_TG_law	Gender Identity Lay (Bolivia)
Brazil_Constitution	Constitution of Brazil
Brazil_SGM_discrimination_2019	Brazilian Supreme Court decision to punish SGM discrimination equivalent to racism
Brazil_SGM_Health_Policy_2013	List of health related SGM policies (Brazil)
Brazil_SGM_laws	List of laws and rights related to SGM from Brazil
Brazil_TG_law_2018	Gender Identity Lay (Brazil)
Chile_Constitution	Constitution of Chile
Chile_Ley_Identidad_de_Genero	Gender Identity Lay (Chile)
Chile_Union_Civil	Chile – civil union law
Colombia_Constitution	Constitution of Colombia
Colombia_Law_SGM_2018	List of laws and rights related to SGM from Colombia
Costa_Rica_Constitution	Constitution of Costa Rica
Costa_Rica_Derechos_LGBTI_2018	List of laws and rights related to SGM from Costa Rica
Costa_Rica_Family_Code	Family law from Costa Rica
Costa_Rica_Gender_Identity	Gender Identity law (Costa Rica)
Cuba_New_Constitution	Constitution of Cuba
Cuba_Penal_Code	Penal Code (Cuba)

Cuba_Work_Legislation_2014	Labor legislation (Cuba)
Dominica_Constitution	Constitution of Dominica
Dominican_Republic_Civil_Code	Civil Code (Dominican Republic)
Dominican_Republic_Constitution_2010	Constitution of Dominican Republic
Dominican_Republic_Penal-Code	Penal Code (Dominican Republic)
Ecuador_Constitution	Constitution of Ecuador
Ecuador_law_anti_discrimination	Legislation related to SGM discrimination (Ecuador)
Ecuador_Same_Sex_Marriage_2019	Legislation that authorizes same-sex marriage in Ecuador
Ecuador_TG_law	Gender Identity Lay (Ecuador)
El_Salvador_Constitution	Constitution of El Salvador
El_Salvador_Discrimination_Law	Legislation related to SGM discrimination (El Salvador)
El_Salvador_Rights_Violation	SGM rights violations (El Salvador)
Grenada_Constitution	Constitution of Grenada
Guatemala_Constitution	Constitution of Guatemala
Guatemala_Gender_Identity_Law	Gender Identity Lay (Guatemala)
Guyana_Constitution	Constitution of Guyana
Haiti_Constitution	Constitution of Haiti
Haiti_Penal_Code	Penal Code (Haiti)
Honduras_Constitution	Constitution of Honduras
Honduras_Constitutional_ban	Honduras constitutional ban of same-sex marriage
Honduras_Decrete_2008	Legislation related to SGM discrimination (Honduras)
Jamaica_Constitution	Constitution of Jamaica
Mexico_Constitution	Constitution of Mexico
Mexico_SGM_laws	Sexual Orientation and gender identity laws (Mexico)
Nicaragua_Codigo_penal	Penal Code (Nicaragua)
Nicaragua_Constitution	Constitution of Nicaragua
Nicaragua_HIV_law	Nicaragua legislation related to HIV/AIDS
Nicaragua_Same-Sex_Constitutional-Ban	Constitutional ban of same-sex marriage (Nicaragua)
Panama_Anti-discrimination-law	Legislation related to SGM discrimination (Ecuador)
Panama_Constitution	Constitution of Panama
Paraguay_Constituition	Constitution of Paraguay
Peru_Constitution	Constitution of Peru
Peru_gender_Violence	Legislation addressing gender-based violence (Peru)
Peru_SGM_anti-discrimination	Legislation related to SGM discrimination (Peru)
Saint_Lucia_Constitution	Constitution of Saint Lucia
St_Kitts_and_Nevis_Constitution	Constitution of Saint Kittis and Nevis
St_Vincent_and_Grenadines_Constitution	Constitution of St Vincent and the Grenadines

Suriname_Constitution	Constitution of Suriname
Trinidad_Tobago_Constitution	Constitution of Trinidad and Tobago
Uruguay_Constitution	Constitution of Uruguay
Uruguay_Gender_Identity	Gender Identity law (Uruguay)
Uruguay_SGM_laws	List of laws and rights related to SGM from Uruguay
Venezuela_CODIGO ORGANICO DE JUSTICIA MILITAR	Military legislation (Venezuela)
Venezuela_Constitution	Constitution of Venezuela
Venezuela_UN_Report	United Nations report about SGM from Venezuela
**Additional selected documents**	**Description**
Amnesty_International_No-safe-place	Report about SGM rights violations in El Salvador, Guatemala and Honduras from Amnesty International
Human_Rights_Watch_2018	Human Rights Watch, World Report (2018)
Human_Rights_Watch_Caribbean	Human Rights Watch. “I Have to Leave to Be Me” Discriminatory Laws against LGBT People in the Eastern Caribbean
IACHR_24-17	IACHR/OAS, Consultee Opinion 24/17
ICCPR	International Covenant on Civil and Political Rights - Office of the High Commissioner for Human Rights
ICESCR	International Covenant on Economic, Social and Cultural Rights - Office of the High Commissioner for Human Rights
ILGA_Sexual_Orientation_Laws_Map_2019	Worldwide map of sexual orientation laws. International Lesbian, Gay, Bisexual, Trans and Intersex Association (ILGA)
ILGA_State_Sponsored_Homophobia_2019	World Report about state-sponsored homophobia. International Lesbian, Gay, Bisexual, Trans and Intersex Association (ILGA)
IofM_LGBT_Health_Report_Brief	Institute of Medicine. Brief Report about the Health of Lesbian, Gay, Bisexual and Transgender People
OAS_LGBTI_Report	IACHR/OAS. Report: Violence against lesbian, gay, bisexual, trans and intersex persons in the Americas. 2015
UDHR	Universal Declaration of Human Rights
UN_Human_Rights_Report_2017	United Nations. Human Rights Report, 2017.
UN_Living_Free_And_Equal	United Nations. Living Free & Equal, 2016
